# ID23-2: an automated and high-performance microfocus beamline for macromolecular crystallography at the ESRF

**DOI:** 10.1107/S1600577522000984

**Published:** 2022-02-22

**Authors:** Max Nanao, Shibom Basu, Ulrich Zander, Thierry Giraud, John Surr, Matias Guijarro, Mario Lentini, Franck Felisaz, Jeremy Sinoir, Christian Morawe, Amparo Vivo, Antonia Beteva, Marcus Oscarsson, Hugo Caserotto, Fabien Dobias, David Flot, Didier Nurizzo, Jonathan Gigmes, Nicolas Foos, Ralf Siebrecht, Thomas Roth, Pascal Theveneau, Olof Svensson, Gergely Papp, Bernard Lavault, Florent Cipriani, Ray Barrett, Carole Clavel, Gordon Leonard

**Affiliations:** a European Synchrotron Radiation Facility, 71 Avenue des Martyrs, F-38000 Grenoble, France; b European Molecular Biology Laboratory, 71 Avenue des Martyrs, 38042 Grenoble, France; c ARINAX, 365 Rue de Corporat, 38430 Moirans, France

**Keywords:** microcrystallography, macromolecular crystallography, MD3Up high-precision multi-axis diffractometer

## Abstract

ID23-2 is a fixed-energy (14.2 keV) microfocus beamline at the European Synchrotron Radiation Facility (ESRF) dedicated to macromolecular crystallography. The optics and sample environment have recently been redesigned and rebuilt to take full advantage of the upgrade of the ESRF to the fourth generation Extremely Brilliant Source (ESRF-EBS). The upgraded beamline now makes use of two sets of compound refractive lenses and multilayer mirrors to obtain a highly intense (>10^13^ photons s^−1^) focused microbeam (minimum size 1.5 µm × 3 µm full width at half-maximum). The sample environment now includes a FLEX-HCD sample changer/storage system, as well as a state-of-the-art MD3Up high-precision multi-axis diffractometer. Automatic data reduction and analysis are also provided for more advanced protocols such as synchrotron serial crystallographic experiments.

## Abstract 2

ID23-2 is a microfocus synchrotron beamline dedicated to macromolcular crystallography at the ESRF-EBS.

## 1. Introduction

Macromolecular microcrystallography is an indispensable tool in modern structural biology. It’s ubiquity has made it easy to forget that the ability to analyze very small crystals was not always so simple. Pioneering studies in macromolecular crystallography (MX) using microbeams were performed on microfocus beamlines such as ESRF ID13 (Cusack *et al.*, 1998 ▸) which laid the groundwork for many of the techniques and instrumentation currently employed today (Perrakis *et al.*, 1999 ▸). At the time, there were no microfocus beamlines dedicated to MX, which was the motivation for the design and construction of the first ‘dedicated’ macromolecular microcrystallography beamline: ID23-2 (Flot *et al.*, 2010 ▸). The design of this beamline emphasized usability and stability in order to make MX microcrystallography accessible to non-experts in microfocus methods. The efficacy of this type of beamline has been well validated, and the early success of ID23-2 has, in some part, been the motivation for the construction and planning of microfocus beamlines at other synchrotron facilities worldwide (Duran *et al.*, 2013 ▸; Evans *et al.*, 2007 ▸; Aragão *et al.*, 2018 ▸; Gu *et al.*, 2021 ▸; Schneider *et al.*, 2021 ▸; Yoder *et al.*, 2010 ▸; Hirata *et al.*, 2010 ▸; Logan *et al.*, 2015 ▸).

A small beam with a large flux density enables several types of experiments. The most obvious of these is of course data collection from very small crystals. This can be in the form of a single microcrystal mounted in a loop or mesh, data collection from multiple microcrystals in a single loop, or even data collections from multiple crystals in a cell (Axford *et al.*, 2014 ▸). Although the ability to use microcrystals offers huge reductions in the resources required to produce larger crystals, the latter case (while exceptional) is illustrative of experiments that are most effectively performed with microbeams. Another example of this type of experiment is the analysis of crystals from native sources, where the amount of protein can be extremely limited – particularly for less abundant proteins (Totir *et al.*, 2012 ▸). Microbeams enable a second type of experiment, which is the rastering of the beam through multiple positions in the crystals, with simultaneous data collection. This type of experiment can be invaluable in determining regions of larger crystals with the best properties [for example, highest resolution, best diffraction spot shape, lowest mosaicity or lowest anisotropy (Bowler *et al.*, 2010 ▸)], and/or locating a crystal in an optically distorted or opaque drop (for example with crystals grown in lipidic cubic phases).

Small beams also facilitate more advanced data collection strategies in which multiple, usually low-completeness, sub-datasets are collected from multiple microcrystals and/or positions on non-microcrystals by rapidly scanning across sample holders and simultaneously collecting data at high frame rates (Liu *et al.*, 2013 ▸; Gati *et al.*, 2014 ▸; Stellato *et al.*, 2014 ▸; Nogly *et al.*, 2015 ▸). These so-called ‘serial crystallography’ experiments can be performed on a wide variety of samples in diverse sample supports and across a wide range of crystal sizes at both cryo and ambient temperatures. They offer a number of potential advantages over traditional ‘one crystal, one sample holder’ experiments, such as the elimination of cryoprotection, the study of crystals in a more ‘native’ state, the removal of the requirement to harvest crystals and the possibility to study dynamic processes. Small-beam serial experiments can range from still images collected from individual crystals to hybrid methods in which small oscillation ranges are collected from crystals. In addition, the possibility of stopped-flow serial crystallography in combination with high-speed detectors has already made the study of some biochemical processes possible (Monteiro *et al.*, 2020 ▸; Beyerlein *et al.*, 2017 ▸; Wang *et al.*, 2014 ▸; Schmidt, 2013 ▸). Finally, theoretical and experimental work has introduced the exciting possibility that smaller beams could in fact produce less radiation damage to macromolecular crystals because of photoelectron escape (Nave & Hill, 2005 ▸; Cowan & Nave, 2008 ▸; Sanishvili *et al.*, 2011 ▸; Dickerson *et al.*, 2020 ▸; Storm *et al.*, 2020 ▸), particularly at high photon energies. All of these aspects of MX microcrystallography, concomitantly with the design of the ESRF-EBS [Extremely Brilliant Source (Raimondi, 2016 ▸)] with its dramatic improvement in brilliance, were the motivations for upgrading the ID23-2 beamline.

## 2. Optical layout of ID23-2

**Figure 1:**
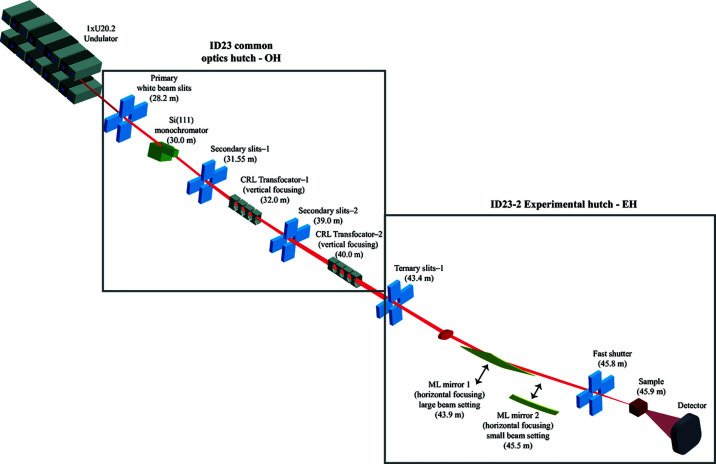
Optical layout of ID23-2. Distances from the source are shown in parentheses. ID23-2 shares a common optical hutch with ID23-1. In the optics hutch (rectangle on the left), energy selection and beam deflection are achieved by the monochromator (green) and vertical focusing is performed by sets of CRLs in transfocators. In the experimental hutch (shown in the rectangle on the right), the beam is then horizontally focused by one of the two multilayer mirrors.

The goals of the optical upgrade of ID23-2 were to provide a smaller beam size in addition to the proven ∼6 µm × 4 µm [H × V, full width at half-maximum (FWHM)] beam size with roughly the same flux. X-rays are produced by a single 1.6 m-long 20.2 mm-period undulator with a minimum gap of 11 mm. The ID23-2 insertion device is located on the upstream segment of a canted straight section [the downstream segment contains the insertion device for beamline ID23-1 (Nurizzo *et al.*, 2006 ▸)]. The beam proceeds through the front-end into the optical hutch, where the beam tails are reduced with a set of high-power primary slits 28.2 m from the source (Fig. 1 ▸). The high-power primary slits are a pair of liquid-cooled copper blocks with a 7 mm × 3 mm (H × V) hole bored through the block. These blocks are mounted on horizontal and vertical translation tables (Flot *et al.*, 2010 ▸). A liquid-nitro­gen-cooled Si(111) monochromator selects a fixed energy of 14.2 keV 30 m from the source for ID23-2 and also deflects the beam laterally, away from the ID23-1 beam originating from the downstream cant, leaving ample room between the two beamlines for downstream optics and sample environments. The monochromator design remained unchanged compared with the original installation (Flot *et al.*, 2010 ▸), with the exception of the introduction of a linear incremental encoder on the Bragg rotation of the monochromator and a fully vacuum-compatible UHV microjack for monochromator Bragg angle fine adjustment.

Vertical and horizontal focusing are decoupled, and are achieved by two transfocators containing 1D beryllium compound refractive lens (CRL) sets (for vertical focus) and two sets of elliptically figured multilayer mirrors (for horizontal focus). Each of these four elements can be translated in and out of the beam depending on the desired beam sizes. The general strategy is to use lenses from the first transfocator in combination with the first mirror to produce the largest beam, and lenses from the second transfocator together with a second mirror for the smallest beam. However, higher aspect ratio beam profiles can be obtained by mixing and matching, and, indeed, using lenses from both transfocators.

## 3. Vertical focusing

**Figure 2:**
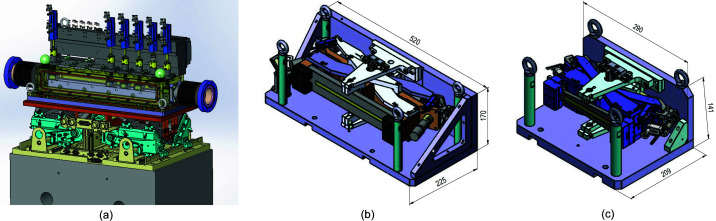
(*a*) Transfocator CAD-drawing. Pneumatic axes (blue) actuate the insertion of lens pack holders into the beam path and onto a high-precision V-rail for mutual lens pack alignment. Alignment (rotation and translation) of the transfocator is achieved through a Q-Sys motion platform (cyan). (*b*) Large-beam and (*c*) small-beam HFM assembly. The mirrors are shown in gray, with the illuminated region in yellow. Dimensions are given in millimetres.

After lateral deflection by the monochromator, the beam passes through a set of JJ X-Ray ib-c30-hv slits (https://www.jjxray.dk/) (31.55 m from the source), and into the first transfocator (32 m from the source). The transfocator assembly is mounted on a Q-sys (http://www.q-sys.eu/) four-axis positioning table and can be controlled via pseudo motors in *BLISS* (*BeamLine Instrument Support Software*), a Python-based open-source ESRF software suite for high-level experimental control (Guijarro *et al.*, 2018 ▸). The transfocator is a standard ESRF design with nine pneumatically actuated axes (Fig. 2 ▸).

**Figure 3:**
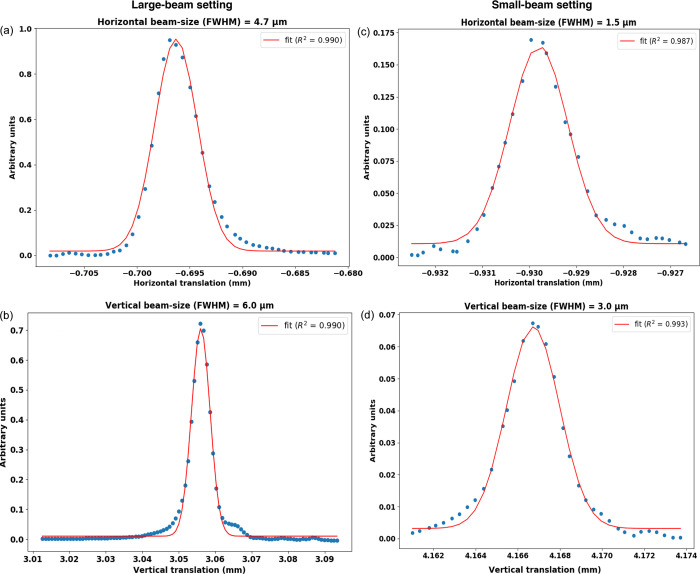
Beam sizes in FWHM, measured by a knife-edge scan of a 5 µm tungsten wire through the X-ray beam (*a*) and (*c*) horizontally or (*b*) and (*d*) vertically with readout from a downstream calibrated PIN diode. Fitting was performed in *PyMCA* (https://github.com/vasole/pymca).

Pinholes of 2 mm diameter are mounted in the first and last positions to facilitate alignment. Different combinations of lenses were mounted in stacks on each pneumatic axis, corresponding to the analytical calculations (Table 1 ▸). Additionally, two larger radius lenses were included in each transfocator to permit fine tuning of the focal distance. After the first transfocator, the beam reaches a second set of slits 39 m from the source. At 40 m, the beam reaches the second transfocator. This is identical to the first, with the exception of the lens configuration (Table 1 ▸). Because lens stacks are pneumatically actuated, the vertical beam sizes at the sample position can be rapidly (<1 s) changed by a simple pulldown menu. The user simply selects the desired vertical beam size, and an internal lookup table contains the corresponding set of lens stacks to insert into the beam. Sizes from the minimum (2.9 µm FWHM) to 40 µm are available without realignment of the beam position. In order to determine the beam size, a 100 µm boron microfilament with a 5 µm tungsten core (Goodfellow, Lille, France) was mounted on the diffractometer and rastered vertically and horizontally through the beam. X-ray intensity was recorded downstream of the wire on a PIN diode (PIN-10DPI, OSI Optoelectronics, Hawthorn, USA). The resulting beam profile obtained after differentiation of the S-shaped raw intensity curve is shown in Figs. 3 ▸(*b*) and 3 ▸(*d*).

## 4. Horizontal focusing

The large-beam horizontally focusing mirror (HFM) is a 240 mm-long bendable [W/B_4_C]_100_ coated multilayer graded mirror with 2.5 nm *d*-spacing, 2.1 m from the sample position and 43.9 m from the source, and provides a 21× demagnification ratio. This mirror works at an 18 mrad incidence angle. Coating and metrology of the mirror were performed at the ESRF. The initially flat mirror is elliptically figured using a dual actuator bender giving a root-mean-square (RMS) slope error (measured by optical metrology) of 0.50 µrad after mounting in the bender. The mirror is mounted in the same mechanical support as previously described (Flot *et al.*, 2010 ▸) and shown in Fig. 2 ▸ (*b*). The bending moments are applied to the mirror via the mechanical support using two stepper motor actuated jacks, and the position is read by two optical encoders (MicroE Mercury series https://www.celeramotion.com/). A large-stroke (35 cm) translation allows the entire mirror in its bender to be translated into and out of the beam (*y* direction). The optimized beam size was measured to be 4.7 µm FWHM as shown in Fig. 3 ▸(*a*), which is the normal operating setting, but unfocused horizontal beam sizes up to 18 µm FWHM are also possible.

The small-beam HFM is a 140 mm-long bendable [W/B_4_C]_100_ laterally graded multilayer mirror with 2.5 nm layer spacing (at the center) and provides a 91× demagnification ratio. Coating and metrology of the mirror were performed at the ESRF. The center of this mirror is 500 mm upstream of the sample position, and 45.5 m from the source, also working at an 18 mrad incidence angle. The mirror mechanics are bent with two vacuum New Focus picomotors. Encoding is facilitated by optical encoders (MicroE Mercury series) [Fig. 2 ▸(*c*)]. The RMS slope error was measured (by optical metrology) to be 0.57 µrad over the central 130 mm (and 0.37 µrad for the central 120 mm) in the bender system. The minimum beam size was measured to be 1.5 µm FWHM [Fig. 3 ▸(*c*)]. Photon flux in the small- and large-beam settings was calculated as per Owen *et al.* (2009 ▸) from diode readings to be 1.5 × 10^13^ and 1.8 × 10^13^ photons s^−1^, respectively, and are unaffected by the vertical beam size.

The same large stroke translation as the larger beam mirror is employed to translate the mirror in and out of the beam. The design of independent vertical and horizontal focusing enables fast switching between large- and small-beam settings by moving the HFMs in/out of the beam path (shown in Fig. 1 ▸). However, in practice, once the *y* position is optimized relative to the incoming beam, the small-beam HFM no longer needs to be moved. Once the first HFM is moved into the beam, the table is translated 54 cm in the *y* direction. This motion is highly repeatable, with the beam position within ±1 µm of the initial position, after the full 54 cm movement, and takes 3 min. The incidence angles of the two mirror systems were deliberately chosen to be the same (18 mrad) so that only a translation of the downstream experimental table between the reflected beams is required, without rotation.

Several devices have been installed for beam alignment and diagnostics. Three sets of motorized translations, containing YAG, diamond or carbon foils, can be inserted into the beam path via *BLISS*. Permanently installed BASLER cameras with visible-light optics can be used to visualize the beam image on fluorescent screens via *LIMA* (Petitdemange *et al.*, 2014 ▸). Diodes, connected to Keithley (Tektronix, Beaverton, OR/USA) pico-ammeters, allow for the alignment of upstream optical elements when the beam viewer axis is moved to the foil-containing position. These beam viewers/diodes are installed in the white beam after the primary slits, immediately after the monochromator, immediately after the first transfocator, and after the second transfocator at 28.80, 31.00, 33.00 and 40.95 m from the source, respectively.

## 5. Sample environment

Changes in temperature can cause the beam and sample to drift in position. In order to improve the thermal stability of the experimental hutch, an ante room was installed to buffer the temperature changes caused by entering the experimental hutch. Additionally, a new, higher capacity air-conditioning unit was installed, with exit grates replaced by fabric ducting, to better distribute the air flow within the hutch, and to reduce air turbulence. Together, these changes improve the thermal stability to ±0.5°C.

**Figure 4:**
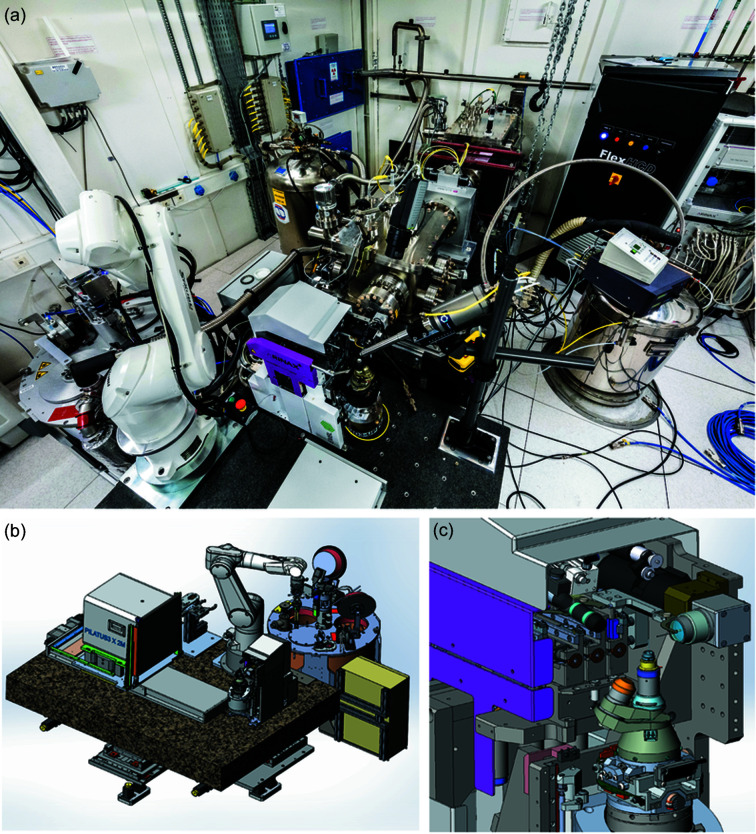
ID23-2 sample environment. (*a*) Photograph of the sample environment (Stef Cande, ESRF), (*b*) MD3Up, detector and FLEX-HCD, (*c*) close-up of MD3Up.

In addition to the selected primary focusing elements, the beam passes through an attenuator block of the same design as on ID30B (McCarthy *et al.*, 2018 ▸), with the same configuration of carbon and aluminium filters (1 and 2 mm carbon; 0.20, 0.35, 0.50, 1.00 and 1.50 mm aluminium) before the first mirror. These attenuators are on a series of pneumatic supports, and are controlled via *BLISS*. In addition, slits have been installed upstream of both mirror systems in order to control the divergence of the reflected beam, and also to optimize mirror bending by rastering a pencil beam across the mirror surface. JJ X-ray slits are installed upstream of the small-beam mirror vessel and a rotary fast shutter (von Stetten *et al.*, 2020 ▸) is attached to the MD3 diffractometer and controlled by the MD3 PMAC controller *via* an ICEPAP motor controller (Janvier *et al.*, 2013 ▸). Sample storage and changing are handled by a FLEX-HCD dewar and sample changer system (Fig. 4 ▸) (McCarthy *et al.*, 2018 ▸). In contrast to other ESRF MX beamlines, with the exception of MASSIF-1, all 23 sample cells are configured to hold Universal Pucks (Uni-Pucks). This allows for up to 368 sample holders to be evaluated before the user or beamline staff need to enter the hutch to reload the HCD. Robust error handling has been designed into both hardware and software, with optical checks for sample presence and alignment on the FLEX-HCD and inductively on the MD3 goniometer head. Dedicated control software and GUI based on *JLib* java toolbox (EMBLEM Technology Transfer GmbH, Heidelberg, Germany; http://software.embl-em.de) manage all FLEX-HCD operations, including sample loading, dewar refilling, puck detection and synchronization between the diffractometer, FLEX robotic arm and the HCD dewar.

An MD3Up diffractometer (ARINAX, Moirans, France; see Fig. 4 ▸) has been installed on the experimental table. This state-of-the-art device offers better than a 200 nm sphere of confusion in diameter, measured optically. MD3Up offers rapid (<1 µm following error at 15 mm s^−1^) vertical and horizontal movements with the sample alignment motors, critical for rapid rastering through samples. An MK3 mini-kappa device (Cipriani *et al.*, 2007 ▸) is also installed, which allows for re-alignment of the crystal – for example to align a long unit cell with the goniometer rotation axis. Because horizontally mounted MK3 devices have a relatively large sphere of confusion, mini-kappa usage was only possible in limited situations with the previous MD2M (horizontal omega axis) diffractometer. Measurement of the sphere of confusion at the sample position with the kappa device in this vertical orientation confirmed no detectable degradation in the sphere of confusion with the kappa unit closed, and only minor degradations at ‘open’ kappa angles [80 nm at *k* = 0° (closed), 110 nm at *k* = 120° and 130 nm at *k* = 180°] and as a result is permanently mounted. Additionally, a crystallization plate gripper is now available on the beamline, compatible with some SBS-formatted crystallization plates. Finally, a DECTRIS Pilatus3 2M with a 450 µm silicon sensor (Dectris AG, Baden-Daettwil, Switzerland) is installed on a motorized translation table, which is driven by an ICEPAP motor controller and is available in the *BLISS* environment (Guijarro *et al.*, 2018 ▸). Detector triggering is controlled by the MD3Up PMAC controller. Detector parameters and image writing are mediated by the *LIMA* software (https://lima1.readthedocs.io).

## 6. Software

The *MXCuBE3* beamline control program (Oskarsson *et al.*, 2018 ▸; Oscarsson *et al.*, 2019 ▸) provides a powerful and intuitive interface for both routine data collections as well as more sophisticated semi and fully automated workflows (Brockhauser *et al.*, 2012 ▸; Zander *et al.*, 2015 ▸). Once collected, data are automatically processed using *XDSAPP*, *GRENADES*, *xia2* and *autoproc* (Vonrhein *et al.*, 2011 ▸; Krug *et al.*, 2012 ▸; Monaco *et al.*, 2013 ▸; Winter *et al.*, 2018 ▸). Furthermore, input files are automatically generated for *XDS* (Kabsch, 2010 ▸). Data collection parameters and processed data are made available with a web interface using the *ISPyB* database (Delagenière *et al.*, 2011 ▸) and EXI front-end (http://exi.esrf.fr/). Data collected with the *mesh and collect* serial crystallography workflow (Zander *et al.*, 2015 ▸) are automatically processed as follows: individual sub-datasets are integrated with *XDS*. Once an initial pass has been completed, the dataset with the highest overall 〈*I*/σ(*I*)〉 is used as the reference dataset, and the *XDS CORRECT* step is re-run for all other datasets using this dataset as the REFERENCE_DATA_SET. The resulting consistently indexed set of partial datasets is then submitted for grouping with *ccCluster* and *CODGAS* (Zander *et al.*, 2016 ▸; Santoni *et al.*, 2017 ▸; Foos *et al.*, 2019 ▸).

Once the data have been integrated, several downstream structure solution pipelines are available. Several sources for molecular replacement search models are used, in two categories. In the first mode, the unit-cell parameters are compared with existing entries in the Protein Data Bank [PDB (Berman *et al.*, 2000 ▸)] using the program *SAUC* (McGill *et al.*, 2014 ▸). Positive matches are downloaded from the PDB. The second category provides significantly more flexibility to the user, and allows the user to define multiple components in the crystal, and/or alternative search models via *EXI*. The models can either be directly uploaded, or a UniProt ID can be provided. In the latter case, the UniProt ID is mapped to existing entries in the PDB and also the EBI Alphafold (Jumper *et al.*, 2021 ▸) database (https://alphafold.ebi.ac.uk/). Molecular replacement is performed in *PHASER* (McCoy *et al.*, 2007 ▸), and the results are uploaded to *ISPyB*. 2*F*_o_ − *F*_c_ and *F*_o_ − *F*_c_ electron density maps are viewable within *EXI* via *UGLYmol* (Wojdyr, 2017 ▸) in a web browser, and all log files can be directly downloaded. Similarly, either individual ligands or collections of ligands can be uploaded to *ISPyB* in SMILES, MOL2 or SDF format, and ligand fitting is performed using *Coot* ‘find ligand’ (Emsley *et al.*, 2010 ▸). If an anomalous signal is detected, the data are submitted to *SHELXC/D/E* (Sheldrick, 2010 ▸; Monaco *et al.*, 2013 ▸) for automatic phasing with experimental electron density maps and partial models available for display and download as above.

## 7. Pre- and post-EBS data statistics

**Figure 5:**
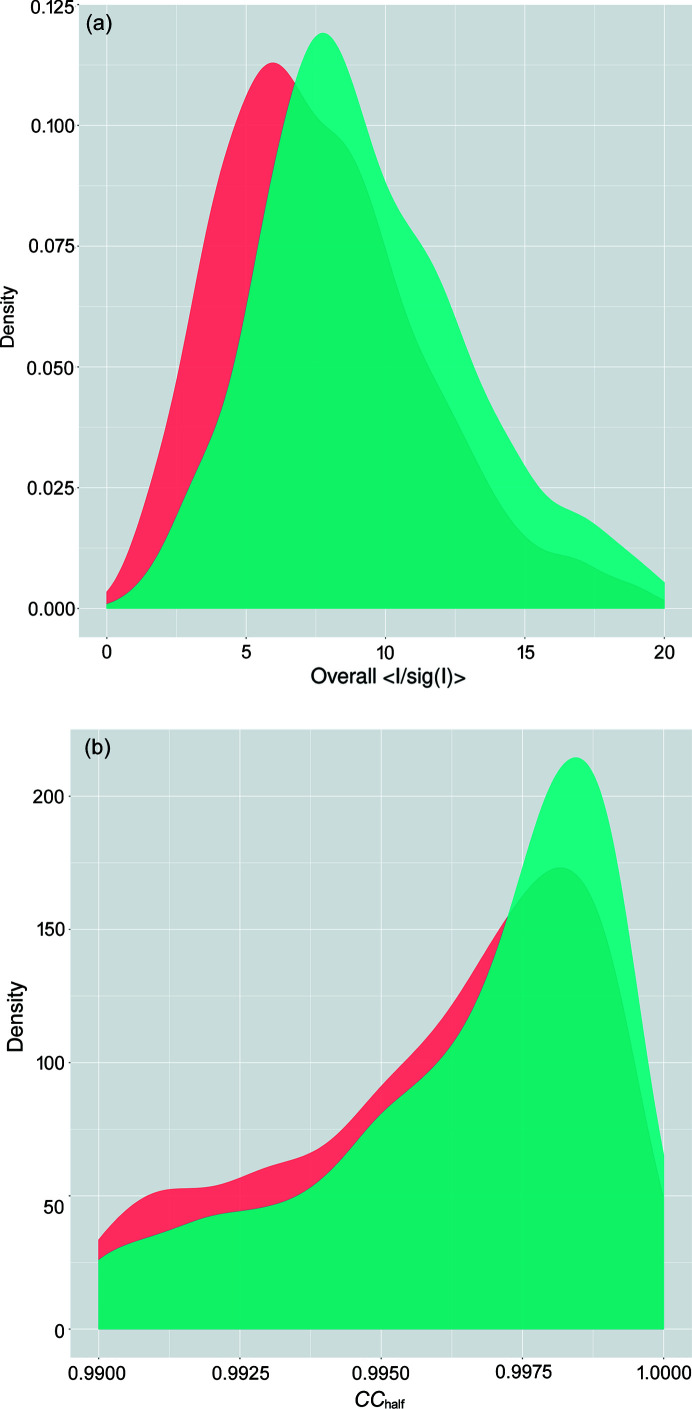
(*a*) Kernel densities of overall 〈*I*/σ(*I*)〉 for the six months of operation preceding the EBS upgrade (red) and six months of operation post-EBS upgrade (green). (*b*) Overall *CC* ^1/2^ values.

The diversity of projects and user workflows make any rigorous statistical comparisons of merging statistics difficult. Nevertheless, the large number of datasets that are automatically processed allows for some comparisons of datasets pre- and post-EBS, provided that caution is used in their (over)interpretation. All beamline upgrades were completed in the middle of 2017, 1.5 years before the EBS upgrade. The six-month periods leading up to the shutdown in 2018, and the first six months of operation in 2021 were chosen as the analysis period. Datasets from ID23-2 were gathered from EXI/ISPyB and filtered to remove screening and poorly indexed/integrated datasets by selecting those with overall completeness >90%, overall *CC* ^1/2^>0.4 and overall *R*_merge_ <0.4. Plots of smoothed kernel density estimates were calculated using the geom_density function of *ggplot2* (https://ggplot2.tidyverse.org/) within *R* (https://www.R-project.org/). This function uses the *R* density (https://www.rdocumentation.org/packages/stats/versions/3.6.2/topics/density) function to produce the kernel density, using a Gaussian kernel and produces curves of frequencies that are less sensitive to histogram bin widths than simple histograms. A significant improvement to the overall 〈*I*/σ(*I*)〉 can be observed after the EBS [Fig. 5 ▸(*a*)]. The mean overall 〈*I*/σ(*I*)〉 values pre- (*n* = 900) and post- (*n* = 924) EBS were 7.75 and 9.78, respectively. A Welch two-sample *t*-test (Welch, 1947 ▸) was performed in *R* (https://www.R-project.org/). This modification of the standard *t*-test does not assume equal variances or sizes between the two populations and yielded a *p*-value of 2.2 × 10^−16^, indicating that the populations are different. While not as dramatic as for 〈*I*/σ(*I*)〉, the *R*_merge_ values also improved significantly (*p*-value of 1.45 × 10^−6^) from pre- to post-EBS with mean values of 22.5 and 19.2%, respectively. Interestingly, the overall *CC* ^1/2^ (Karplus & Diederichs, 2012 ▸) values were not significantly different between the two populations (*p*-value of 0.928), although there was a very modest shift in the distribution of *CC* ^1/2^ values [Fig. 5 ▸(*b*)]

## 8. Example data collections

**Figure 6:**
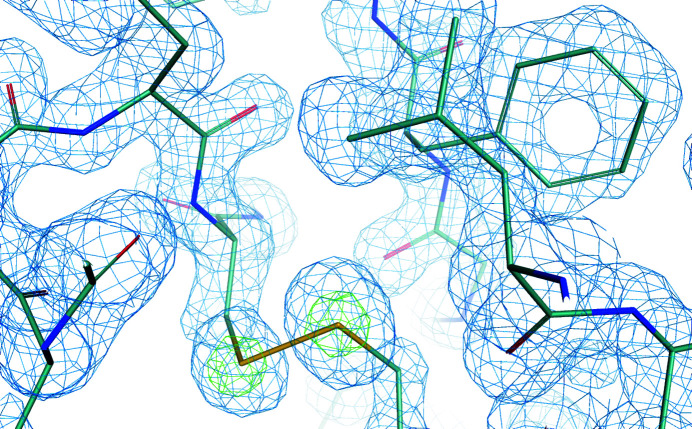
Experimental electron density (blue) from native sulfur anomalous phasing of cubic insulin on data collected at *E* = 14.2 keV/0.873 Å. Density is contoured at 1.5σ above the mean electron density value. Model-phased anomalous difference density is shown in green, contoured at 10σ above the mean electron density value. This figure was generated in *PyMOL* (Schroedinger).

The high flux density of ID23-2 makes analysis of microcrystals the most common use for the beamline. However, the microbeam is also routinely used to attain higher multiplicity datasets from larger single crystals. This is typically achieved by collecting datasets from multiple positions on a crystal, or by using helical data collections (Flot *et al.*, 2010 ▸). One example of the latter can be seen in Table 2 ▸, taken with the small-beam setting (1.5 µm × 3 µm FWHM). In this example, a large (300 µm × 50 µm × 50 µm) cubic insulin crystal was used to collect 3600 frames helically with heavy attenuation, yielding a flux of 1 × 10^11^ photons s^−1^ at an oscillation step of 0.1° with 30 ms exposures, in 7/8 multibunch mode with 200 mA ring current. Despite the extremely non-optimal energy, these data were of sufficient quality that experimental phases could be determined from the very weak sulfur anomalous signal [*f*′′ at 14.2 keV = 0.2e^−^, estimated anomalous signal of 0.4% (http://bmsc.washington.edu/scatter)]. *SHELXC/D/E* (Sheldrick, 2010 ▸) and *CRANK2* (Pannu *et al.*, 2011 ▸) were used for phasing and density improvement, followed by cycles of manual building in *Coot* (Emsley *et al.*, 2010 ▸) and refinement in *REFMAC5* (Murshudov *et al.*, 2011 ▸). Substructure search was performed in *SHELXD* with the DSUL 3 keyword specified, using data to 2.5 Å resolution and yielded a substructure solution with a CFOM of 42.2, *CC*_all_ of 24.75 and *CC*_weak_ of 17.5 after 4000 cycles. Strong contrast was observed between the two hands in *SHELXE*, with a final *CC* of the partial model of 41.5% after two automatic building cycles. Calculation of a model phased anomalous difference map in *ANODE* (Thorn & Sheldrick, 2011 ▸) revealed peak heights of S positions of 14.9, 14.9, 12.9, 12.9, 12.7 and 12.6 for CYS 19B, CYS 20A, CYS 7A, CYS11A, CYS 7B and CYS 6A, respectively. Experimental electron density is shown in Fig. 6 ▸. The refined structure has been deposited in the PDB with the accession code 7qgf, and the raw images have been made available on Zenodo (https://zenodo.org/record/5761266).

A second example of achieving higher multiplicity and signal-to-noise compared with what is possible from a single crystal is the use of the *mesh and collect* SSX approach (Zander *et al.*, 2016 ▸). The crystallization of insulin microcrystals was set up using a 24-well sitting drop plate. A volume of 1 µl human insulin (Sigma I2643) at 20 mg ml^−1^ concentration was mixed with 1 µl of the reservoir solution. The reservoir solution was composed of 450 m*M* Na_2_HPO_4_ and 10 m*M* EDTA at pH = 10.4. The crystals were grown within a day of crystallization setup. A slurry of ∼15 µm^3^ insulin microcrystals were harvested on a 700 µm-diameter micromesh loop with 10 µm openings (Mitegen) and cooled directly in the cryostream. The *mesh and collect* workflow (Zander *et al.*, 2015 ▸) was used to collect 228 partial datasets of 100 × 0.1° oscillations, with the beam attenuated to 3.5 × 10^11^ photons s^−1^ with 40 ms exposures. Data were indexed and integrated in *XDS* (Kabsch, 2010 ▸) from within *GrenADES* (Monaco *et al.*, 2013 ▸). Partial datasets were grouped with *CODGAS* (Zander *et al.*, 2016 ▸) and the merging statistics of the best group (containing 142 datasets) are shown in Table 2 ▸.

## 9. Conclusions

ID23-2 is a fixed-energy energy microcrystallography beamline dedicated to macromolecular crystallography with over 1310 PDB depositions, 1264 publications and 14 patents attributed to it. Recently, the beamline was almost completely rebuilt, dramatically improving the beamline capabilities. Taken together with the ESRF-EBS upgrade, this beamline offers unique capabilities which will enable future advances in structural biology.

## Supplementary Material

PDB reference: 7qgf

## Figures and Tables

**Table 1:** Summary of the beamline parameters, including source, optical configuration, X-ray beam characteristics and the experimental setup for ID23-2 at the ESRF

Beamline name	ID23-2	
Source type	1 × 1.6 m 20.2 mm-period undulator	
Horizontal emittance (pm rad)	132	
Vertical emittance (pm rad)	5	
Monochromator	Si(111)	
Energy (keV)	14.2	
Horizontal photon source size (µm RMS)	30.3	
Vertical photon source size (µm RMS)	4.5	
Horizontal photon divergence (µrad RMS)	6.9	
Vertical photon divergence (µrad RMS)	5.6	

Vertical focusing		
Focusing elements	1D CRLs in two transfocators	
Lens material	Be	
Transfocator	Large beam	Small beam
Axis 1 (mm)	2 mm alignment pinhole	2 mm alignment pinhole
Axis 2 [No. of lenses × radius (µm)]	1 × 200	1 × 200
Axis 3 (µm)	5 × 200	12 × 200
Axis 4 (µm)	1 × 500	1 × 500
Axis 5 (µm)	1 × 300	1 × 300
Axes 6–7 (µm)	Unused	Unused
Axis 8 (µm)	2 mm alignment pinhole	2 mm alignment pinhole
Transfocator efficiency (%)	93	88
Vertical demagnification ratio	2.4×	8×

Horizontal focusing		
Mirror assembly	Large beam	Small beam
Horizontal focusing mirror (HFM)	Bendable tangential mirror	Bendable tangential mirror
Incident angle on HFM (mrad)	18	18
Slope error of HFM (µrad RMS)	0.50	0.57
Horizontal demagnification ratio	21×	91×

Sample position		
Beam size (H × V, FWHM) (µm)	4.7 × 6.0	1.5 × 3.0
Flux (photons s^–1^)	1.8 × 10^13^	1.5 × 10^13^
Diffractometer	MD3Up	
Sample mounting	FLEX HCD with rapid-exchange single and double grippers	
Detector type	CMOS Hybrid Pixel-Array	
Detector model	Dectris PILATUS3 X 2M (450 µm Si sensor)	

**Table 2:** Data collection and refinement statistics Statistics for the highest-resolution shell are shown in parentheses. For the *mesh and collect* data, the average cell edge and range are provided. A refinement was not performed for these data.

	Cubic insulin helical	Cubic insulin *mesh and collect*
Wavelength (Å)	0.873	0.873
No. of crystals	1	142
Resolution range (Å)	39.28–1.203 (1.246–1.203)	32.07–1.750 (1.80–1.75)
Space group	*I*2_1_3	*I*2_1_3
Unit cell	*a* = *b* = *c* = 78.50	*a* = *b* = *c* = 78.47 (78.27–78.73)
	α = β = γ = 90	α = β = γ = 90
Total reflections	2269388 (146102)	1257717 (95649)
Unique reflections	25126 (2395)	15814 (1184)
Multiplicity	90.3 (58.7)	79.53 (80.78)
Completeness (%)	99.45 (95.30)	100 (99.7)
〈*I*/σ(*I*)〉	30.66 (1.26)	25.48 (1.36)
Wilson *B*-factor	17.03	13.18
*R*_meas_	0.106 (2.697)	0.155 (4.143)
*CC*_1/2_	1 (0.565)	1 (0.685)
Anomalous correlation (inner)	2	3
SigAno	0.816	0.842
Reflections used in refinement	25010 (2395)	–
Reflections used for *R*_free_	1264 (129)	–
*R*_work_	0.0468 (0.0768)	–
*R*_free_	0.0442 (0.0755)	–
*CC*_work_	0.951 (0.736)	–
*CC*_free_	0.959 (0.706)	–
No. of non-hydrogen atoms	459	–
Macromolecules	396	–
Solvent	63	–
Protein residues	50	–
RMS (bonds)	0.016	–
RMS (angles)	1.66	–
Ramachandran favored (%)	100.00	–
Ramachandran allowed (%)	0.00	–
Ramachandran outliers (%)	0.00	–
Rotamer outliers (%)	0.00	–
Clashscore	3.90	–
Average *B*-factor	22.63	–
Macromolecules	19.55	–
Solvent	42.04	–
