# ID23-2: an automated and high-performance microfocus beamline for macromolecular crystallography at the ESRF. Corrigendum

**DOI:** 10.1107/S1600577522002818

**Published:** 2022-03-14

**Authors:** Max Nanao, Shibom Basu, Ulrich Zander, Thierry Giraud, John Surr, Matias Guijarro, Mario Lentini, Franck Felisaz, Jeremy Sinoir, Christian Morawe, Amparo Vivo, Antonia Beteva, Marcus Oscarsson, Hugo Caserotto, Fabien Dobias, David Flot, Didier Nurizzo, Jonathan Gigmes, Nicolas Foos, Ralf Siebrecht, Thomas Roth, Pascal Theveneau, Olof Svensson, Gergely Papp, Bernard Lavault, Florent Cipriani, Ray Barrett, Carole Clavel, Gordon Leonard

**Affiliations:** a European Synchrotron Radiation Facility, 71 Avenue des Martyrs, F-38000 Grenoble, France; b European Molecular Biology Laboratory, 71 Avenue des Martyrs, 38042 Grenoble, France; c ARINAX, 365 Rue de Corporat, 38430 Moirans, France

**Keywords:** microcrystallography, macromolecular crystallography, MD3Up high-precision multi-axis diffractometer

## Abstract

A revised version of Table 2 of Nanao *et al.* [ *J. Synchrotron Rad.* (2022). **29**, 581–590] is provided.

## Abstract 2

Corrections to Table 2 of Nanao *et al.* [ *J. Synchrotron Rad.* (2022). **29**, 581–590] are reported.

Some of the values reported in Table 2 ▸ of Nanao *et al.* (2022 ▸) were found to be incorrect. The full correct table is shown below.

## Figures and Tables

**Table 2:** Data collection and refinement statistics Statistics for the highest-resolution shell are shown in parentheses. For the *MeshAndCollect* data, the average cell edge and range are provided. A refinement was not performed for these data.

	Cubic insulin helical	Cubic insulin *MeshAndCollect*
Wavelength (Å)	0.873	0.873
No. of crystals	1	142
Resolution range (Å)	39.28–1.203 (1.246–1.203)	32.07–1.750 (1.80–1.75)
Space group	*I*2_1_3	*I*2_1_3
Unit cell		
*a*, *b*, *c* (Å)	78.50	78.47 (78.27–78.73)
α, β, γ (°)	90	90
Total reflections	2269388 (146102)	1257717 (95649)
Unique reflections	25126 (2395)	15814 (1184)
Multiplicity	90.3 (58.7)	79.53 (80.78)
Completeness (%)	99.45 (95.30)	100 (99.7)
〈*I*/σ(*I*)〉	30.66 (1.26)	25.48 (1.36)
Wilson *B* factor	17.03	13.18
*R*_meas_	0.106 (2.697)	0.155 (4.143)
CC_1/2_	1 (0.565)	1 (0.685)
Anomalous correlation (inner)	2	3
SigAno	0.816	0.842
Reflections used in refinement	23764 (1661)	–
Reflections used for *R*_free_	1264 (89)	–
*R*_work_	0.150 (0.297)	–
*R*_free_	0.164 (0.319)	–
CC_work_	0.952	–
CC_free_	0.948	–
No. of non-hydrogen atoms	457	–
Macromolecules	335	–
Solvent	122	–
Protein residues	50	–
RMS (bonds)	0.021	–
RMS (angles)	2.362	–
Ramachandran favored (%)	100.00	–
Ramachandran allowed (%)	0.00	–
Ramachandran outliers (%)	0.00	–
Rotamer outliers (%)	0.00	–
Clashscore	3.90	–
Average *B* factor	23.51	–
Macromolecules	20.76	–
Solvent	41.56	–
